# High-Intensity Statin vs. Low-Density Lipoprotein Cholesterol Target for Patients Undergoing Percutaneous Coronary Intervention: Insights From a Territory-Wide Cohort Study in Hong Kong

**DOI:** 10.3389/fcvm.2021.760926

**Published:** 2021-10-28

**Authors:** Andrew Kei-Yan Ng, Pauline Yeung Ng, April Ip, Chung-Wah Siu

**Affiliations:** ^1^Cardiac Medical Unit, Grantham Hospital, Wong Chuk Hang, Hong Kong, SAR China; ^2^Department of Adult Intensive Care, Queen Mary Hospital, Hong Kong, SAR China; ^3^Division of Respiratory and Critical Care Medicine, Department of Medicine, Li Ka Shing Faculty of Medicine, The University of Hong Kong, Hong Kong, SAR China; ^4^Department of Medicine, Queen Mary Hospital, The University of Hong Kong, Hong Kong, SAR China

**Keywords:** percutaneous coronary intervention, dyslipidemia, statin, low density lipoprotein cholesterol, major adverse cardiac events, all-cause mortality, myocardial infarction, stroke

## Abstract

**Background:** Different guidelines recommend different approaches to lipid management in patients with atherosclerotic cardiovascular disease. We aim to determine the best strategy for lipid management in Asian patients undergoing percutaneous coronary intervention (PCI).

**Method:** This was a retrospective cohort study conducted in patients who underwent first-ever PCI from 14 hospitals in Hong Kong. All participants either achieved low-density lipoprotein cholesterol (LDL-C) target of <55 mg/dl with ≥50% reduction from baseline (group 1), or received high-intensity statin (group 2), or both (group 3) within 1 yr after PCI. The primary endpoint was a composite outcome of all-cause mortality, myocardial infarction, stroke, and any unplanned coronary revascularization between 1 and 5 yr after PCI.

**Results:** A total of 8,650 patients were analyzed with a median follow-up period of 4.2 yr. After the adjustment of baseline characteristics, complexity of PCI and medications prescribed and the risks of the primary outcome were significantly lower in group 2 (hazard ratio [HR], 0.82; 95% confidence interval [CI], 0.74–0.93, *P* = 0.003) and group 3 (HR, 0.75; 95% CI, 0.62–0.90; *P* = 0.002). The primary outcome occurred at similar rates between group 2 and group 3.

**Conclusions:** Use of high intensity statin, with or without the attainment of guidelines recommended LDL-C target, was associated with a lower adjusted risk of MACE at 5 yr, compared with patients who attained LDL-C target without high intensity statin.

## Background

Deposition of low-density lipoprotein cholesterol (LDL-C) within the arterial wall is a key initiating and propagating event in atherogenesis (1). Numerous randomized controlled trials (RCT) have established the pivotal role of 3-hydroxy-3-methyl-glutaryl-coenzyme A reductase inhibitors (statins) in cardiovascular risk reduction for patients with established coronary artery disease (2, 3). However, there are different approaches in its clinical application. The 2013 American College of Cardiology/American Heart Association (ACC/AHA) guideline recommended high intensity statin without specifying LDL-C targets for patients with established cardiovascular disease (4). In the 2018 revision, the ACC/AHA conjoined with other American societies continued the class I recommendation for routine high-intensity statins, with options of adjunctive lipid-lowering therapy in selected subgroups (5). In contrast, the 2019 European Society of Cardiology/European Atherosclerosis Society (ESC/EAS) guideline recommended an LDL-C target of <55 mg/dl (<1.4 mmol/L) and a 50% LDL-C reduction from baseline for those with established cardiovascular disease which could be achieved by maximally tolerated statin and other lipid-lowering therapies (6).

Due to genetic polymorphism, the plasma levels of statin and its metabolites are generally doubled in Asians as compared with Caucasians (7, 8). Several studies have observed no significant outcome difference with high-intensity statins as compared with moderate intensity statins in Asian patients, (9–12) raising questions whether routine high-intensity statins are needed (13). However, one study showed benefits of high-intensity statins in Asians who attained a less contemporary LDL-C target of <70 mg/dl (<1.8 mmol/L) (14). There were no data comparing the effects of the ACC/AHA vs. ESC/EAS guidelines, especially for Asians. We aimed to determine the best approach to lipid management for Asian patients after percutaneous coronary intervention (PCI).

## Methods

### Study Population and Design

Adult patients (18 yr of age or older) who underwent first-ever PCI between January 1, 2004, and December 31, 2017, at any of the 14 public hospitals in Hong Kong and entered the territory-wide PCI Registry were reviewed. Patient characteristics, exposures, and outcomes were retrieved from the Clinical Data and Analysis Reporting System (CDARS). The study was approved by the Institutional Review Board of the University of Hong Kong/Hospital Authority. The requirement for written informed consent was waived due to the retrospective nature of the study.

We included all patients who survived for at least 365 days after PCI. Patients with outcome events within 365 days were excluded to avoid reverse causality. Patients were excluded if they did not fulfill either the intensity criteria or target criteria as specified below.

### Definitions of Exposure and Outcome Variables

The target criteria were fulfilled if LDL-C was reduced to <55 mg/dl for at least one measurement between 0 and 365 days after PCI, together with ≥50% reduction from baseline if the patient was not previously on statins. The intensity criteria were fulfilled if the patients were prescribed high-intensity statins at any time between 0 and 365 days after PCI. High intensity statin was defined as atorvastatin ≥40mg per day, rosuvastatin ≥20 mg per day, or simvastatin ≥80 mg per day, in accordance with ACC/AHA guideline (remarks: simvastatin was not considered high intensity statin after 2011) (4, 5). Patients were grouped according to the lipid management strategy into three groups. Group 1 included patients fulfilling the target criteria only, group 2 included those fulfilling the intensity criteria only, and group 3 included those fulfilling both target and intensity criteria.

The primary endpoint was a major adverse cardiac event (MACE), defined as a composite outcome of all-cause mortality, non-fatal myocardial infarction, stroke, or any unplanned coronary revascularization, in a time-to-first-event analysis up to 5 yr after PCI. The secondary endpoints were individual components of the primary endpoint, and a composite outcome of all-cause mortality, non-fatal myocardial infarction (MI), and stroke. Detailed definitions are shown in the Supplementary Appendix.

### Statistical Analysis

All analyses were performed with prespecified endpoints and statistical methods. Unadjusted analyses were performed using Chi-square tests for categorical variables and ANOVA tests for continuous variables. Pair-wise comparisons between the three exposure groups were performed for each endpoint. Cox regression analysis was performed to evaluate the independent relationship between lipid management strategy and clinical outcomes, adjusting for potential confounders selected *a priori* based on published data and biological plausibility. Variables adjusted in the Cox model were sex, age, tobacco use, diabetes mellitus, hypertension, dyslipidemia, cerebrovascular disease, peripheral vascular disease, chronic obstructive pulmonary disease, previous myocardial infarction, previous coronary artery bypass surgery, history of heart failure, atrial fibrillation or flutter, baseline anemia (last known hemoglobin before PCI < 13 g/dl for men, or <12 g/dl for women), renal insufficiency (estimated glomerular filtration rate [eGFR] <60 ml/min/m^2^), indication for PCI (stable CAD, unstable angina, nonST elevation myocardial infarction [NSTEMI], and ST elevation myocardial infarction [STEMI]), number of coronary arteries affected, angiographic success, aspirin on discharge, P2Y12 inhibitors on discharge, beta-blocker on discharge, angiotensin blockade on discharge, and PCI period (before and after 2013ACC/AHA guideline) (4). The comorbidities were recorded as a part of mandatory input for the registry. The laboratory results, medication prescriptions, and outcomes were extracted from the CDARS.

### Sensitivity Analyses

We examined a more stringent criteria, such that only patients who fulfilled criteria measured at both periods of 0–180 days and 181–365 days after PCI were considered as criteria fulfillment, and repeated the analysis examining the outcomes occurring between 365 days and 5 yr. Next, we assumed that all our patients belonged to the “very high risk” subgroup according to the 2018 ACC/AHA guidelines, (5) repeated the primary analysis after excluding those who did not achieve 50% reduction in LDL-C (if newly started on statin after PCI), and did not achieve an LDL-C of <70 mg/dl (<1.8 mmol/L) in group 2. In addition, we constructed a propensity score for the likelihood of fulfillment of either target criteria (i.e., group 1) or intensity criteria (i.e., group 2) using logistic regression with the variables in the primary regression model. Matched pairs were generated by 1:1 propensity-score-matching using a caliper of 0.05. Outcomes between the matched pairs were compared.

The complete case method was adopted to address missing data in the primary statistical analysis. To test the robustness of our results, the regression analysis was repeated with the entire cohort using the technique of multiple imputations by chained equations.

### Exploratory Analyses

We studied the effect modifications on the relationship between lipid management strategy (group 2 vs. group 1) and primary outcome by introducing interaction terms to the Cox regression model. These included sex, age >65, diabetes mellitus, eGFR <60 ml/min/m^2^, and acute coronary syndrome.

Data management and statistical analyses were performed in Stata software, version 16 (StataCorp LP). For each endpoint, the Bonferroni–Holm method was used to control for multiple comparisons to maintain a family-wise type I error rate of 0.05 (i.e., statistical significance achieved if *P*-value < 0.0167 for the lowest *P*-value; < 0.025 for the second-lowest *P*-value; < 0.05 for the third-lowest *P*-value) (15).

## Results

### Patients and Characteristics

**Figure 1:**
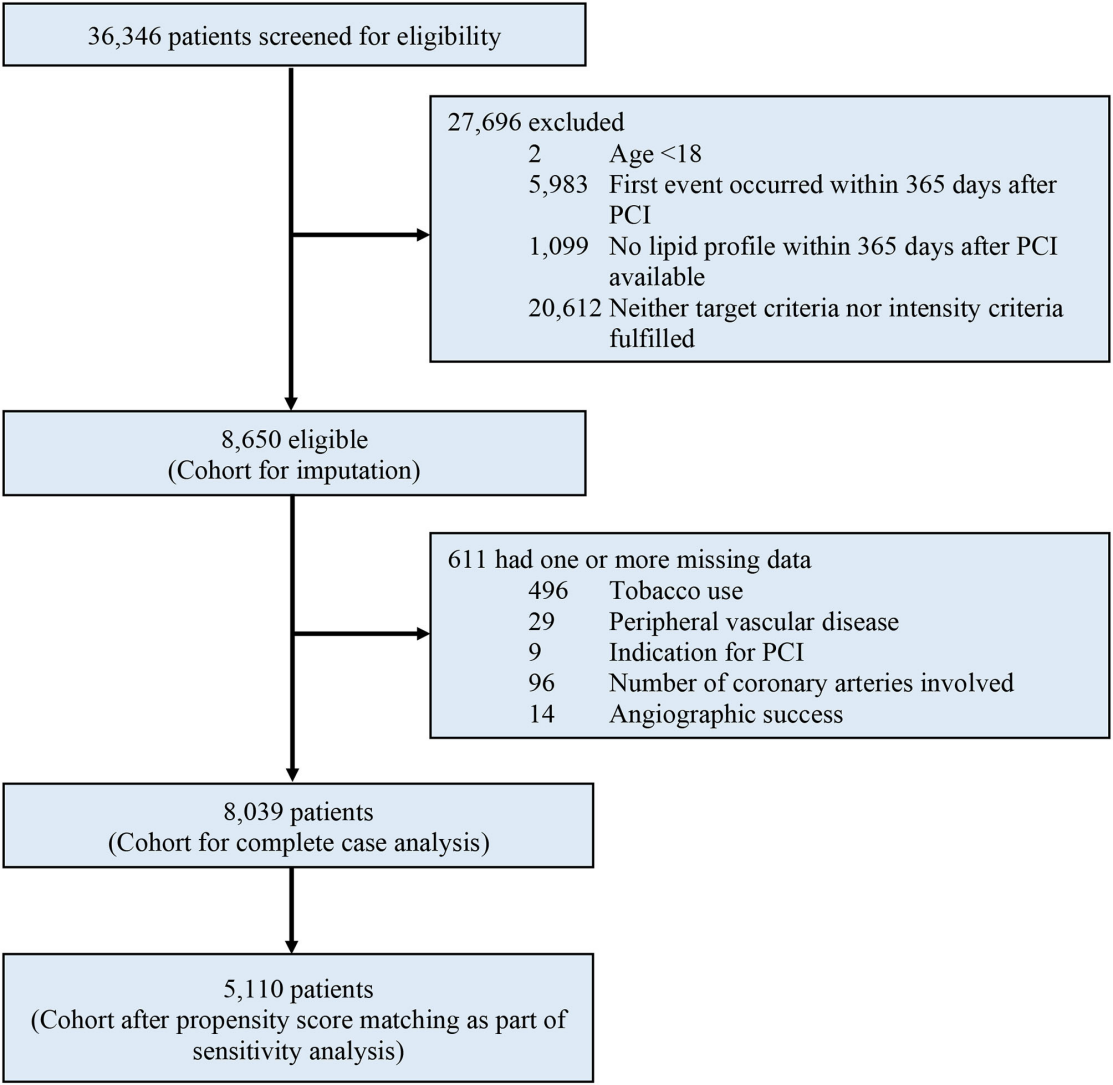
Study profile. eGFR, estimated glomerular filtration rate; PCI, percutaneous coronary intervention.

Between January 2004 and December 2017, a total of 36,346 patients were considered for inclusion, and 27,696 (76.2%) were excluded due to any of the following exclusion criteria: age younger than 18 yr, MACE occurred within 365 days after PCI, orno available lipid profile within 365 days after PCI, or fulfilled neither target nor intensity criteria. Of the remaining 8,650 patients analyzed, a total of 611 (7.1%) were excluded from the complete case analysis due to missing values in any of the variables used in the Cox regression model (Figure 1). The mean age was 63.6 ± 11.5. The cohort consisted of 1,829 (21.1%) female patients and 8,112 (93.8%) were Chinese. A total of 3,832, 3,512, and 1,306 patients were included in group 1, group 2, and group 3 respectively. Table 1 shows the baseline and characteristics of the study population. Patients in group 1 were older, more frequently had diabetes, hypertension, previous myocardial infarction, heart failure, atrial fibrillation or flutter, chronic kidney disease, and anemia. Patients in group 2 and 3 more frequently had PCI done after 2013. Table 2 shows the lipid profile and lipid-lowering medications of the study population. The mean LDL-C at PCI and at one yr, and also the lowest LDL-C within the first yr was higher in group 2. The absolute reduction in LDL-C at one yr was higher in group 2 and group 3.

**Table 1:** Baseline characteristics of patients.

**Characteristics**	**Group 1** **(Target criteria only)**	**Group 2** **(Intensity criteria only)**	**Group 3** **(Both criteria)**	***P*** **value**
N	3,832	3,512	1,306	
Female	924 (24.1%)	680 (19.4%)	225 (17.2%)	<0.001
Age, mean (SD)	66.5 (11.4)	60.8 (10.8)	62.7 (11.3)	<0.001
Chinese	3,648 (95.2%)	3,234 (92.1%)	1,230 (94.2%)	<0.001
Tobacco use	1,402/3569 (39.3%)	1,704/3,356 (50.8%)	570/1,229 (46.4%)	<0.001
Diabetes mellitus	1,753 (45.7%)	938 (26.7%)	458 (35.1%)	<0.001
Hypertension	2,687 (70.1%)	1,864 (53.1%)	784 (60.0%)	<0.001
Cerebrovascular disease	395 (10.3%)	221 (6.3%)	93 (7.1%)	<0.001
Peripheral vascular disease	56/3,819 (1.5%)	33/3,502 (0.9%)	11/1,300 (0.8%)	0.058
Chronic obstructive pulmonary disease	81 (2.1%)	44 (1.3%)	26 (2.0%)	0.015
Previous myocardial infarction	498 (13.0%)	329 (9.4%)	92 (7.0%)	<0.001
Previous CABG	82 (2.1%)	76 (2.2%)	26 (2.0%)	0.93
Congestive heart failure	324 (8.5%)	151 (4.3%)	52 (4.0%)	<0.001
Atrial fibrillation or flutter	250 (6.5%)	84 (2.4%)	39 (3.0%)	<0.001
eGFR <60 ml/min/m^2^	875 (22.8%)	384 (10.9%)	194 (14.9%)	<0.001
Anemia^*^	1,363 (35.6%)	881 (25.1%)	369 (28.3%)	<0.001
PCI indication				<0.001
Stable CAD	849/3,824 (22.2%)	641/3,511 (18.3%)	219 (16.8%)	
Unstable angina	874/3,824 (22.9%)	709/3,511 (20.2%)	209 (16.0%)	
NSTEMI	1,642/3,824 (42.9%)	1,558/3,511 (44.4%)	579 (44.3%)	
STEMI	459/3,824 (12.0%)	603/3,511 (17.2%)	299 (22.9%)	
Number of arteries involved				<0.001
One vessel disease	1,828/3,808 (48.0%)	1,460/3,459 (42.2%)	576/1,287 (44.8%)	
Two vessel disease	1,235/3,808 (32.4%)	1,183/3,459 (34.2%)	439/1,287 (34.1%)	
Three vessel disease	745/3,808 (19.6%)	816/3,459 (23.6%)	272/1,287 (21.1%)	
Angiographic success	3,741/3,819 (98.0%)	3,442/3,511 (98.0%)	1,275/1,306 (97.6%)	0.67
Aspirin on discharge	3,731 (97.4%)	3,424 (97.5%)	1,279 (97.9%)	0.52
P2Y12 inhibitor on discharge	3,790 (98.9%)	3,487 (99.3%)	1,301 (99.6%)	0.030
Beta-blocker on discharge	2,977 (77.7%)	2,602 (74.1%)	1,005 (77.0%)	0.001
Angiotensin blockade on discharge	2,119 (55.3%)	1,603 (45.6%)	609 (46.6%)	<0.001
PCI done in 2013 or later	2,401 (62.7%)	2,870 (81.7%)	1,199 (91.8%)	<0.001

*SD, standard deviation; CABG, coronary artery bypass surgery; eGFR, estimated glomerular filtration rate; PCI, percutaneous coronary intervention; CAD, coronary artery disease*.

* *Anemia: Hemoglobin <13g/dl for men, <12g/dl for women*.

**Table 2:** Lipid profile (values in mg/dl) and lipid lowering treatment.

**Characteristics**	**Group 1** **(Target criteria only)**	**Group 2** **(Intensity criteria only)**	**Group 3** **(Both criteria)**	***P*** **value**
N	3,832	3,512	1,306	
LDL-C at PCI, mean (SD)	78.1 (34.4)	127.6 (46.4)	95.5 (42.5)	<0.001
LDL-C at 1 year, mean (SD)	53.0 (17.0)	89.7 (30.6)	49.9 (17.8)	<0.001
Lowest LDL-C between 0–365 days, mean (SD)	44.0 (8.5)	84.9 (28.7)	42.6 (8.7)	<0.001
Reduction in LDL-C at 1 year, mean (IQR)	21.0 (−2.6–53.2)	31.2 (6.2–69.6)	45.3 (13.3–75.0)	<0.001
% Reduction in LDL-C at 1 year, mean (IQR)^#^	30.2% (5.3–53.0%)	27.0% (6.5–46.0%)	49.5% (22.8–62.7%)	<0.001
Triglyceride at PCI, mean (SD)	148.8 (129.3)	155.9 (121.4)	167.4 (155.0)	<0.001
Triglyceride at 1 year, mean (SD)	135.5 (113.4)	129.3 (93.9)	125.8 (93.0)	0.011
Reduction in triglyceride at 1 year, mean (95% CI)	10.0 (−18.6–44.3)	17.6 (−10.7–54)	17.7 (−8.0–62.0)	<0.001
Atorvastatin on discharge	857 (22.4%)	2,125 (60.5%)	914 (70.0%)	<0.001
Rosuvastatin on discharge	369 (9.6%)	802 (22.8%)	321 (24.6%)	<0.001
Simvastatin on discharge	2,461 (64.2%)	539 (15.3%)	59 (4.5%)	<0.001
Other statins on discharge	11 (0.3%)	4 (0.1%)	0 (0%)	<0.001
Non-statin therapy within 1 year	41 (1.1%)	236 (6.7%)	54 (4.1%)	<0.001
Ezetimibe	41 (1.1%)	235 (6.7%)	53 (4.1%)	<0.001
PCSK9 inhibitor	0 (0.0%)	1 (<1%)	1 (0.1%)	0.28

*LDL-C, low-density lipoprotein cholesterol; PCI, percutaneous coronary intervention; SD, standard deviation; IQR, inter-quartile range*.

# *Not equivalent to a reduction from baseline measurement before statin therapy since some patients were already on statin before PCI*.

### Primary Outcome

**Figure 2:**
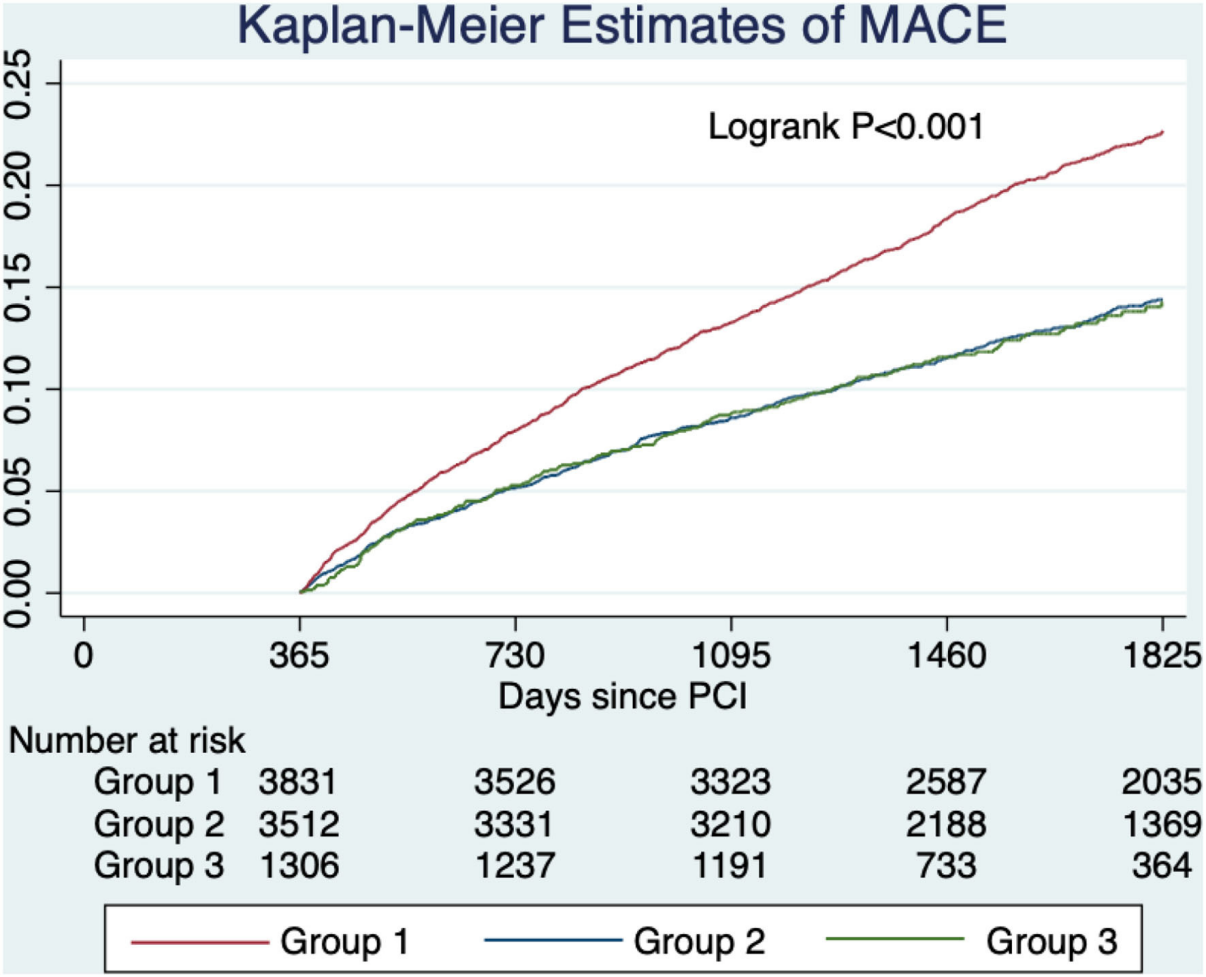
Unadjusted estimated probabilities of major adverse cardiovascular events stratified by lipid management strategy. Group 1 (target criteria only) had a higher risk of MACE compared with group 2 (intensity criteria only) and group 3 (both criteria).

After a median follow-up of 4.2 yr, the primary outcome of MACE has developed in 811 (21.2%) patients in group 1 in 448 (12.8%) in group 2, and in 160 (12.3%) in group 3 (Table 3 and Figure 2). In adjusted analysis with covariates listed in the Method section, the risks of MACE at 5 yr were significantly lower in group 2 (hazard ratio [HR], 0.82; 95% confidence interval [CI], 0.72–0.93, *P* = 0.003) and group 3 (HR, 0.75; 95% CI, 0.62–0.90; *P* = 0.002) (Table 4). The primary outcome occurred at similar rates between group 2 and group 3.

**Table 3:** Unadjusted annualized risks (95% confidence interval) of primary and secondary outcomes.

**Outcomes**	**Group 1** **(Target criteria only)**	**Group 2** **(Intensity criteria only)**	**Group 3** **(Both criteria)**
Primary			
Major adverse cardiovascular events	6.58% (6.14–7.05%)	4.03% (3.68–4.42%)	4.04% (3.46–4.71%)
Secondary			
All-cause mortality	2.93% (2.65–3.23%)	1.22% (1.04–1.44%)	1.51% (1.18–1.93%)
Myocardial infarction	2.15% (1.91–2.42%)	1.39% (1.19–1.63%)	1.37% (1.05–1.78%)
Stroke	1.31% (1.13–1.52%)	0.74% (0.60–0.91%)	0.82% (0.59–1.15%)
Unplanned revascularization	1.71% (1.50–1.95%)	1.60% (1.39–1.85%)	1.43% (1.11–1.85%)
Composite of all–cause mortality, myocardial infarction and stroke	5.36% (4.97–5.78%)	2.92% (2.62–3.25%)	3.09% (2.59–3.68%)

**Table 4:** Adjusted hazard ratios of primary and secondary outcomes.

**Outcomes**	**Group 1** **(Target criteria only)**	**Group 2** **(Intensity criteria only)**		**Group 3** **(Both criteria)**	
		**Hazard Ratio (95% CI)**	***P* Value**	**Hazard Ratio (95% CI)**	***P*** **Value**
Primary					
Major adverse cardiovascular events	Reference	0.82 (0.72–0.93)	0.003^*^	0.75 (0.62–0.90)	0.002^*^
		Reference		0.92 (0.76–1.11)	0.38
Secondary					
All–cause mortality	Reference	0.77 (0.62–0.95)	0.015^*^	0.77 (0.58–1.02)	0.07
		Reference		1.00 (0.73–1.37)	0.99
Myocardial infarction	Reference	0.87 (0.70–1.09)	0.23	0.82 (0.60–1.13)	0.22
		Reference		0.94 (0.68–1.30)	0.72
Stroke	Reference	090 (0.67–1.20)	0.46	0.99 (0.67–1.46)	0.96
		Reference		1.11 (0.74–1.66)	0.63
Unplanned revascularization	Reference	0.87 (0.70–1.09)	0.22	0.75 (0.54–1.04)	0.084
		Reference		0.86 (0.63–1.19)	0.37
Composite of all-cause mortality, myocardial infarction and stroke	Reference	0.82 (0.71–0.95)	0.010^*^	0.78 (0.64–0.96)	0.020^*^
		Reference		0.95 (0.77–1.18)	0.66

* *Significant P valve(s) after using the Bonferroni-Holm method to control for multiple comparisons*.

### Secondary Outcomes

Patients in group 2 had a lower risk of all-cause mortality compared with group 1 (HR, 0.77; 95% CI, 0.62–0.95; *P* = 0.015). The composite endpoint of all-cause mortality, MI, and stroke was lower in group 2 (HR, 0.82; 95% CI, 0.71–0.95; *P* = 0.010) and group 3 (HR, 0.78; 95% CI, 0.64–0.96; *P* = 0.020). Other secondary outcomes were not significantly different across pair-wise comparisons using the Bonferroni–Holm method to control for multiple comparison. Detailed results are shown in Table 4.

### Sensitivity Analyses

After reclassification of patients using more stringent criteria, there were 1,816 patients in group 1, 3,071 patients in group 2, and 538 patients in group 3. Patients in group 2 had a lower risk of MACE compared with group 1 (HR, 0.74; 95% CI, 0.63–0.86; *P* < 0.001), but patients in group 3 had similar risks of MACE compared with group 1 (HR, 0.73; 95% CI, 0.55–0.97; *P* = 0.027) and group 2 (HR, 0.99; 95% CI, 0.75–1.31, *P* = 0.95).

Next, we excluded 256 patients from group 2 because they achieved less than 50% reduction in LDL-C (if newly started on statin after PCI) or failed to reduce LDL-C below 70 mg/dL (1.8 mmol/L). The risks of MACE remained significantly lower in group 2 (HR, 0.82; 95% CI, 0.72–0.94, *P* = 0.003) and group 3 (HR, 0.75; 95% CI, 0.62–0.90; *P* = 0.002) when compared with group 1. The risks of MACE were similar between group 2 and group 3 (HR, 0.92; 95% CI, 0.76–1.11, *P* = 0.37). These findings were consistent with the primary analysis.

**Figure 3:**
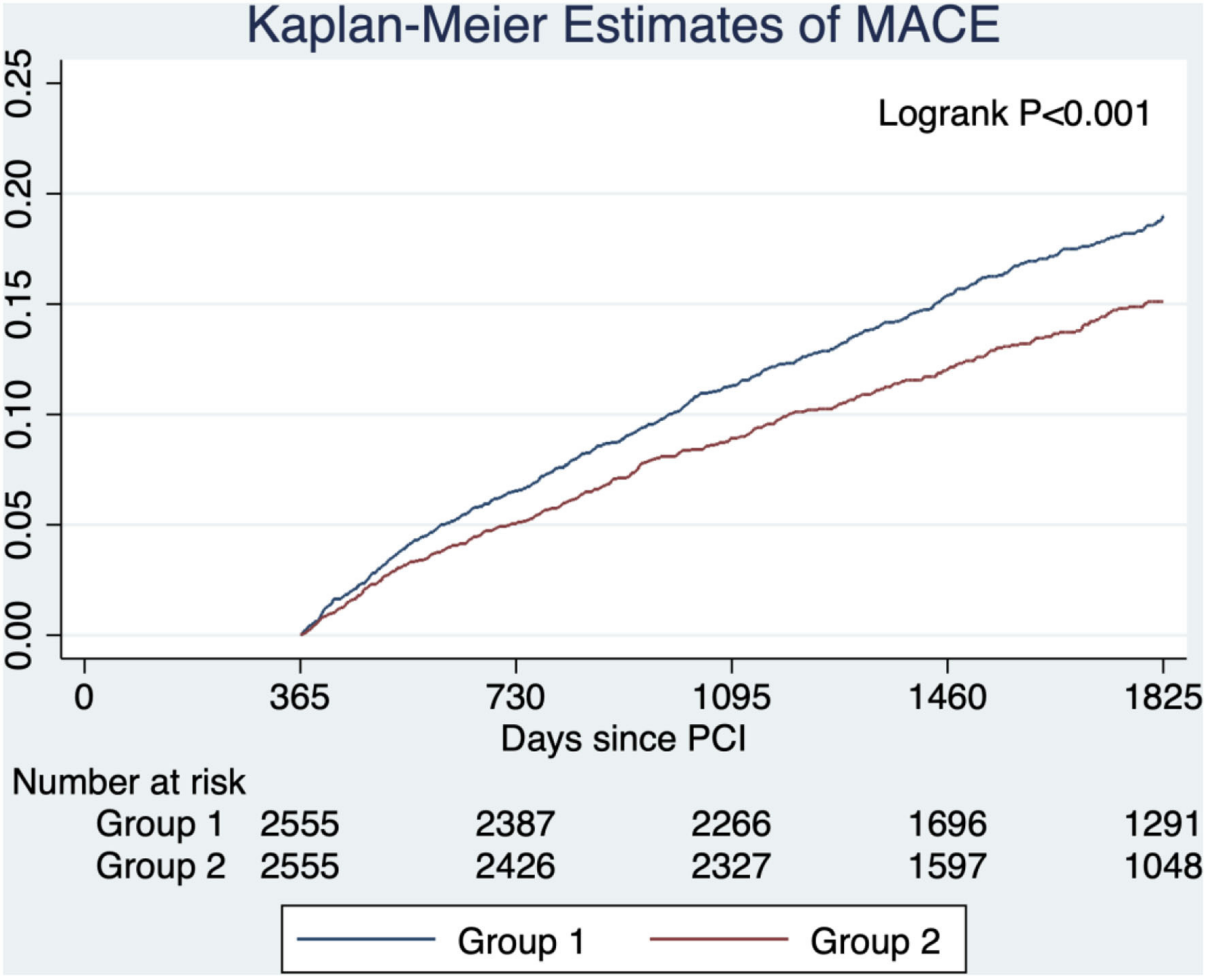
Estimated probabilities of major adverse cardiovascular events stratified by lipid management strategy after propensity score matching. Group 1 (target criteria only) had a higher risk of MACE compared with group 2 (intensity criteria only) in the propensity score-matched cohort.

After propensity score matching, 5,110 patients (2,555 pairs) in group 1 and group 2 were matched. The baseline characteristics were well-balanced with standardized differences of <0.1 in all variables except age (Supplementary Table 1 in the Supplementary Appendix). The risk of MACE was lower in group 2 compared with group 1 (HR, 0.78; 95% CI, 0.68–0.90; *P* < 0.001) (Figure 3).

A total of five variables in the Cox regression model had missing data. Multiple imputation was conducted, and the imputed cohort included all 611(7.1%) patients who were excluded due to missing values in any of the variables used in the model. In the imputed dataset, the risks of MACE were significantly lower in group 2 (HR, 0.82; 95% CI, 0.72–0.93; *P* = 0.001) and group 3 (HR, 0.73; 95% CI, 0.62–0.88; *P* = 0.001) compared with group 1, but the risks of MACE were similar between group 2 and group 3 (HR, 1.22; 95% CI, 0.75–1.08, *P* = 0.26). These findings were consistent with the complete case cohort.

### Exploratory Analyses

**Figure 4:**
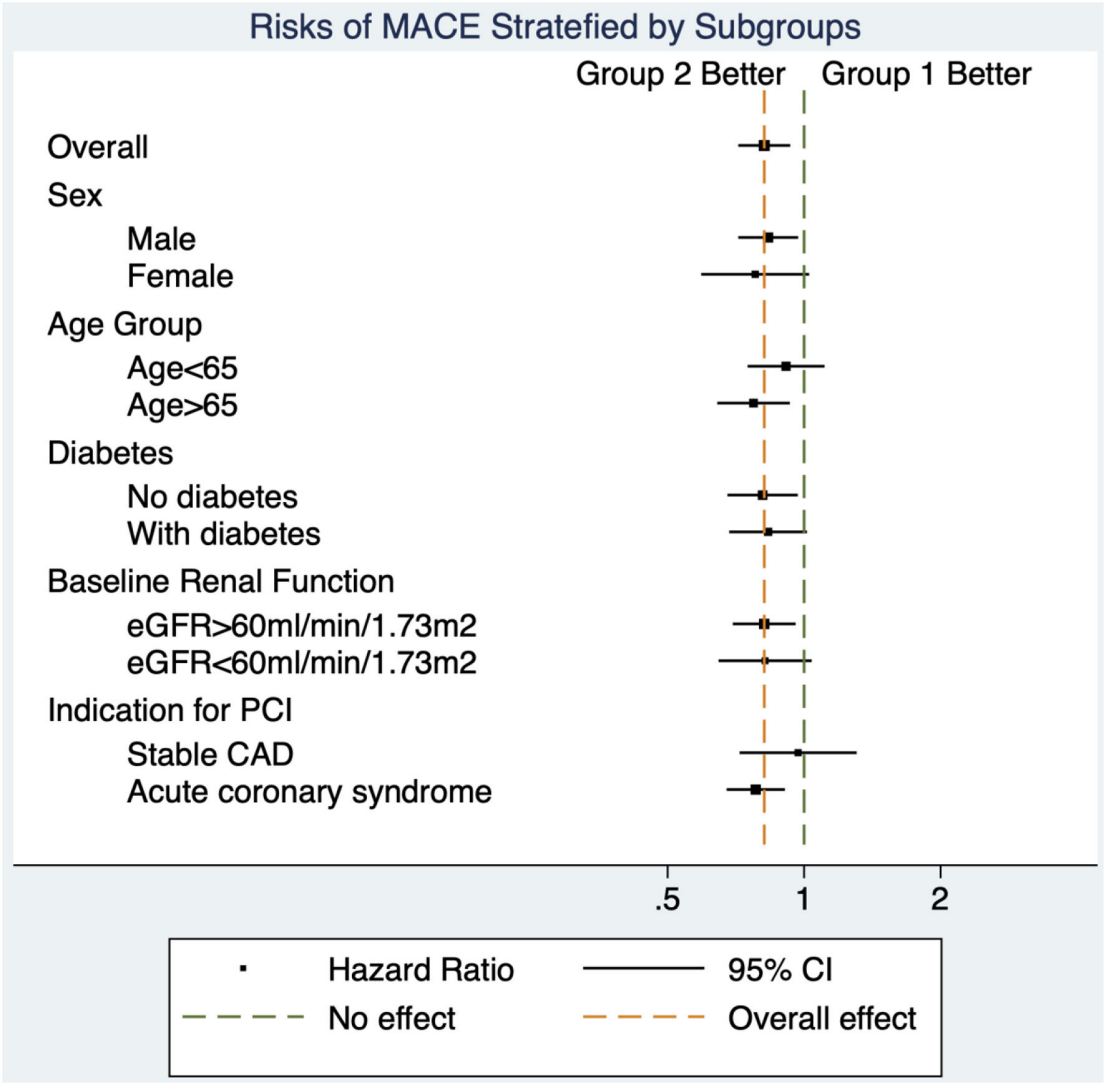
Forest plot for subgroup analyses. The decreased risks of major adverse cardiovascular events in group 2 (intensity criteria only) compared with group 1 (target criteria only) were similar across all subgroups.

The benefits of high-intensity statin were consistent across all subgroups. There was no significant interaction between lipid management strategy and predefined subgroups on the primary outcome (Table 5 and Figure 4).

**Table 5:** Major adverse cardiovascular events in group 2 (intensity criteria only) compared with group 1(target criteria) in predefined subgroups.

**Subgroup**	**Hazard Ratio** **(95% confidence interval)**	***P*** **value for Interaction**
Sex		0.67
Male	0.83 (0.72–0.97)	
Female	0.78 (0.59–1.03)	
Age Group		0.43
Age <65	0.91 (0.75–1.11)	
Age>65	0.77 (0.64–0.93)	
Diabetes		0.50
No diabetes	0.81 (0.68–0.97)	
With diabetes	0.83 (0.68–1.01)	
Baseline Renal Function		0.59
eGFR>60ml/min/1.73m2	0.62 (0.70–0.96)	
eGFR <60ml/min/1.73m2	0.82 (0.65–1.04)	
Indication for PCI		0.24
Stable coronary artery disease	0.97 (0.72–1.31)	
Acute coronary syndrome	0.78 (0.67–0.91)	

*eGFR, estimated glomerular filtration rate; PCI, percutaneous coronary intervention*.

## Discussion

In this cohort of 8,650 adult patients who underwent a first-ever PCI, we showed that high-intensity statins, with or without attainment of LDL-C target in accordance to the latest guideline, (6) were associated with a lower adjusted risk of MACE and all-cause mortality at 5 yr, compared with patients who attained LDL-C target without high-intensity statins. Our data suggests that a strategy of routine high intensity statin may be more suited to the Asian population.

An extensive body of evidence from RCT has shown that statins can reduce cardiovascular events, with greater benefits derived from higher intensity statins (2, 3). All, except one RCT, either compared a fixed-dose statin with placebo (or no treatment) or compared statins at different intensities. The one exception was a RCT designed to evaluate the effect of dose-adjustment to achieve certain LDL-C target in patients with ischemic stroke, (16) but cardiac outcomes were not examined. The 2013 ACC/AHA guideline had recommended the use of high-intensity statins for all patients with atherosclerotic cardiovascular disease (ASCVD), and deemphasized targeting specific LDL-C levels (4). This was a significant departure from other societal guidelines, e.g., Adult Treatment Panel III Guidelines (17) and National Lipid Association Recommendations (18). Although the updated 2018 ACC/AHA guideline includes a goal of 50% reduction in LDL-C levels, routine high-intensity statins remain as the guiding principle of lipid management (5). In contrast, the 2019 ESC/EAS guideline takes a different approach and retains a goal approach to lipid management, recommending a LDL-C target of <55 mg/dl with a 50% reduction from baseline for patients with ASCVD (6). It interprets the totality of evidence as a reflection that cardiovascular risk reduction is proportional to the absolute reduction in LDL-C levels, and that benefits related to LDL-C reduction are not specific to statin therapy. Each of these two approaches has its own merits and short-comings. Routine high-intensity statins for secondary prevention is in line with the large body of evidence showing that the benefits of statin are similar across all levels of baseline LDL-C without any threshold (3). However, lack of emphasis on LDL-C target may make it difficult for clinicians to incorporate the newer effective, non-statin-lipid lowering therapies, (19–21) particularly as an add-on therapy in patients who can tolerate high-intensity statins (22–24).

To our best knowledge, there are no published data comparing these approaches at the most contemporary recommendations. One study used a previous LDL-C target (<70 mg/dL or >50% reduction from baseline level) and found that high-intensity statins were associated with a lower risk of MACE (14). Another study showed that on-treatment, LDL-C level was an independent predictor of MACE, but statin intensity was not (25). Our data suggested that Asians would benefit from high-intensity statins, regardless of attainment of LDL-C target. Importantly, we observed a reduction in mortality which is considered as the important outcome from a perspective of a patient.

There are several plausible explanations. First, the absolute reduction in LDL-C was greater in the high-intensity statin groups (group 2 and 3), and it is likely that the beneficial effects was mediated by LDL-C reduction. Large meta-analyses have concluded that each unit reduction in LDL-C was associated with similar relative risk reduction of MACE without any threshold level (3, 26). Collectively, the LDL-C hypothesis and “the lower the better” principle withstood the test of time (27). Second, statins have several beneficial lipid-independent (pleotropic) effects on atherosclerotic lesions, including favorable effects on endothelial function, modulation of inflammatory response, inhibition of coagulation cascade, and stabilization of plaques (28–31). Pathogenic investigations and clinical trials have suggested that these pleotropic effects of statins are dose-dependent (30, 32, 33). Third, triglyceride levels were lower with a greater degree of reduction in the high intensity statin groups, lending an alternative mechanism to decreasing cardiovascular risk (34).

Due to genetic polymorphism, the plasma levels of statin can be doubled in Asians as compared with Caucasians (7, 8). Asians typically achieve similar LDL-C reduction at lower statin doses (35, 36). Previous observational studies in various Asian populations did not demonstrate superior cardiovascular outcomes with high-intensity statins after PCI and acute myocardial infarction, (9–12) thereby resulting in vastly different statin-prescribing practices. In Japan, for instance, none of the statins is approved at high intensity doses (37). The majority of Asian societal guidelines still recommend a LDL-C target of 70–100 mg/dL for secondary prevention, substantially looser than the ESC/EAS guideline (38–40).

In future guideline revisions, emphasis should be made on either routine high-intensity statins or, if a target should be recommended for practical purposes, on absolute or percentage reduction of LDL-C, as opposed to a universal target for patients with ASCVD. From a practical perspective, 56.7% of the screened patients in our study, unfortunately, received care that was adherent to neither of the two major societal guidelines (Figure 1). This translational gap called for methods that can effectively increase guideline adherence. Further studies are needed to understand the effects of statin adherence, cholesterol variability, and applicability in primary prevention or other subgroups such as elderly or diabetics, (41–43) although we did not observe any effect modification in our subgroup analysis.

This study has some limitations. First, the observational nature of the study conferred risks of unmeasured confounding and bias. However, the large population-based sample size allowed extensive adjustment for potential confounders that may affect lipid management strategy and outcomes, and the findings were consistent with multiple sensitivity analyses. Our data was retrieved from a population-based electronic database with minimal loss to follow up and complete information on laboratory results and subsequent events. All information was recorded *a priori*, thus minimizing the selection, information, and recall biases. Second, we only included patients who had first-ever PCI and survived 365 days, and so the findings may not be applicable to those with recurrent coronary stenosis. We also could not evaluate the effects of high intensity statin initiated before PCI (44, 45). Third, this was an as-treated analysis, and there may be unexplored reasons behind the eventual lipid management strategy.

## Conclusion

Among patients who underwent a first-ever PCI in Hong Kong, high-intensity statins, with or without the attainment of guideline-recommended LDL-C target, were associated with a lower adjusted risk of MACE at 5 yr, compared with patients who attained LDL-C target without high-intensity statins.

## Data Availability Statement

The raw data supporting the conclusions of this article will be made available by the authors, without undue reservation.

## Ethics Statement

The studies involving human participants were reviewed and approved by Institutional Review Board of the University of Hong Kong/Hospital Authority Hong Kong West Cluster. Written informed consent for participation was not required for this study in accordance with the national legislation and the institutional requirements. Written informed consent was not obtained from the individual(s) for the publication of any potentially identifiable images or data included in this article.

## Author Contributions

AN and C-WS were responsible for the conception and design of the study. AN analyzed the data collected by AI. AN interpreted the data. AN and PN drafted the manuscript. All the authors revised and approved the final manuscript, and are accountable for the accuracy and integrity of the study.

## Conflict of Interest

The authors declare that the research was conducted in the absence of any commercial or financial relationships that could be construed as a potential conflict of interest.

## Publisher's Note

All claims expressed in this article are solely those of the authors and do not necessarily represent those of their affiliated organizations, or those of the publisher, the editors and the reviewers. Any product that may be evaluated in this article, or claim that may be made by its manufacturer, is not guaranteed or endorsed by the publisher.
