# Supplementary material for: High-Intensity Statin vs. Low-Density Lipoprotein Cholesterol Target for Patients Undergoing Percutaneous Coronary Intervention: Insights From a Territory-Wide Cohort Study in Hong Kong
Source: Front Cardiovasc Med. 2021 Oct 28;8:760926. doi: 10.3389/fcvm.2021.760926 (PMC8581611; doi:10.3389/fcvm.2021.760926)
Supplement: Supplementary file 1 [file Data_Sheet_1.docx]

**Supplementary Appendix**

**ENDPOINT DEFINITIONS**

**Death**

Deaths is classified as cardiovascular or non-cardiovascular. The cause of death will be determined by the principal condition that resulted in the death, not the immediate mode of death. Managing physicians will utilize all available information provided, along with clinical expertise, in their adjudication of the cause of death.

**Cardiovascular death**

Death due to cardiovascular causes. They include:

- death from acute myocardial infarction and its complications (e.g., arrhythmia, sudden arrest, heart failure)
- sudden cardiac death
- death from heart failure
- death from stroke
- death caused by complications of cardiovascular procedures
- death from cardiovascular hemorrhage (e.g., intracranial hemorrhage, non-procedural or non-traumatic vascular rupture (e.g., aortic aneurysm), or hemorrhage causing cardiac tamponade)
- death from other cardiovascular causes not included in the above categories but with a specific, known cardiovascular cause (e.g., pulmonary embolus or peripheral arterial disease)

**BASELINE VARIABLES DEFINITIONS**

**Estimated glomerular filtration rate**

Estimated glomerular filtration rate (eGFR) is calculated based on MDRD equation, expressed as:

186 × (Creatinine/88.4) - 1.154 × (Age) - 0.203 × (0.742 if female) × (1.210 if black)

where Creatinine is expressed in μmol/L.

**Anemia**

Anemia is defined as hemoglobin <13g/dL for men and hemoglobin <12g/dL for women.

**PCI urgency**

- Elective: Patient cardiac status has been stable in the days or weeks before the operation. The procedure can be deferred without increased risk of compromised cardiac outcome.
- Urgent: Procedure required during the same hospitalization to minimize chances of clinical deterioration or adverse outcome. Clinical conditions include (but are not limited to) acute or worsening chest pain, acute or worsening HF, acute MI, critical coronary stenosis, IABP support, UA with intravenous nitroglycerin, and rest angina.
- Emergency: Procedure required because of ongoing, refractory (difficult, complicated, and/or unmanageable), unrelenting cardiac compromise, with or without hemodynamic instability, and not responsive to any form of therapy except PCI.

Tables

Table S1. Baseline characteristics of patients in group 1 and group 2 after propensity score matching.

| Characteristics | Group 1  (Target criteria only) | Group 2  (Intensity criteria only) | *P* value | Standardized difference |
| --- | --- | --- | --- | --- |
| *N* | 2555 | 2555 |  |  |
| Female | 582 (22.8%) | 543 (21.3%) | 0.19 | 0.037 |
| Age, mean (SD) | 63.755855 (11.360712) | 62.075663 (10.893622) | <0.001 | 0.151 |
| Tobacco use | 1117 (43.7%) | 1184 (46.3%) | 0.060 | -0.052 |
| Diabetes mellitus | 907 (35.5%) | 819 (32.1%) | 0.009 | 0.073 |
| Hypertension | 1615 (63.2%) | 1492 (58.4%) | <0.001 | 0.099 |
| Cerebrovascular disease | 218 (8.5%) | 189 (7.4%) | 0.13 | 0.042 |
| Peripheral vascular disease | 36 (1.4%) | 28 (1.1%) | 0.31 | 0.028 |
| Chronic obstructive pulmonary disease | 45 (1.8%) | 38 (1.5%) | 0.44 | 0.022 |
| Previous myocardial infarction | 254 (9.9%) | 249 (9.7%) | 0.81 | 0.007 |
| Previous CABG | 55 (2.2%) | 50 (2.0%) | 0.62 | 0.014 |
| Congestive heart failure | 132 (5.2%) | 127 (5.0%) | 0.75 | 0.009 |
| Atrial fibrillation or flutter | 90 (3.5%) | 73 (2.9%) | 0.18 | 0.038 |
| eGFR < 60ml/min/m^2^ | 383 (15.0%) | 329 (12.9%) | 0.029 | 0.061 |
| Anemia* | 743 (29.1%) | 680 (26.6%) | 0.049 | 0.055 |
| PCI indication |  |  | 0.081 | 0.073 |
| Stable CAD | 540 (21.1%) | 509 (19.9%) |  |  |
| Unstable angina | 564 (22.1%) | 527 (20.6%) |  |  |
| NSTEMI | 1108 (43.4%) | 1118 (43.8%) |  |  |
| STEMI | 343 (13.4%) | 401 (15.7%) |  |  |
| Number of arteries involved |  |  | 0.30 | -0.043 |
| One vessel disease | 1182 (46.3%) | 1133 (44.3%) |  |  |
| Two vessel disease | 839 (32.8%) | 850 (33.3%) |  |  |
| Three vessel disease | 534 (20.9%) | 572 (22.4%) |  |  |
| Angiographic success | 2505 (98.0%) | 2505 (98.0%) | 1.00 | <0.001 |
| Aspirin on discharge | 2485 (97.3%) | 2489 (97.4%) | 0.73 | -0.010 |
| P2y12 inhibitor on discharge | 2532 (99.1%) | 2535 (99.2%) | 0.65 | -0.013 |
| Beta-blocker on discharge | 1943 (76.0%) | 1900 (74.4%) | 0.16 | 0.039 |
| Angiotensin blockade on discharge | 1310 (51.3%) | 1234 (48.3%) | 0.033 | 0.001 |
| PCI done in 2013 or later | 1890 (74.0%) | 1986 (77.7%) | 0.002 | -0.088 |
